# Multimorbidity: The Brazilian Longitudinal Study of Aging (ELSI-Brazil)

**DOI:** 10.11606/S1518-8787.2018052000637

**Published:** 2018-10-25

**Authors:** Bruno Pereira Nunes, Sandro Rogério Rodrigues Batista, Fabíola Bof de Andrade, Paulo Roberto Borges de Souza, Maria Fernanda Lima-Costa, Luiz Augusto Facchini

**Affiliations:** I Universidade Federal de Pelotas. Faculdade de Enfermagem. Departamento de Enfermagem em Saúde Coletiva. Pelotas, RS, Brasil; II Universidade Federal de Goiás. Faculdade de Medicina. Goiânia, GO, Brasil; III Fundação Oswaldo Cruz. Instituto René Rachou. Programa de Pós-Graduação em Saúde Coletiva. Belo Horizonte, MG, Brasil; IV Fundação Oswaldo Cruz. Instituto René Rachou. Núcleo de Estudos em Saúde Pública e Envelhecimento. Belo Horizonte, MG, Brasil; VI Fundação Oswaldo Cruz. Instituto de Comunicação e Informação Científica e Tecnológica em Saúde. Rio de Janeiro, RJ, Brasil; VII Universidade Federal de Pelotas. Faculdade de Medicina. Departamento de Medicina Social. Pelotas, RS, Brasil

**Keywords:** Aged, Multimorbidity, Comorbidity, Health Surveys, Idoso, Multimorbidade, Comorbidade, Inquéritos Epidemiológicos

## Abstract

**OBJECTIVE:**

To evaluate the occurrence and factors associated with multimorbidity among Brazilians aged 50 years and over.

**METHODS:**

This is a cross-sectional study in a nation-based cohort of the non-institutionalized population in Brazil. Data were collected between 2015 and 2016. Multimorbidity was assessed from a list of 19 morbidities, which were categorized into ≥ 2 and ≥ 3 diseases. The analysis included the calculation of frequencies and the most frequent 10 pairs and triplets of combinations of diseases. The crude and adjusted analyses evaluated the demographic, socioeconomic, behavioral, and contextual variables (area of residence, geopolitical region, and coverage of the Family Health Strategy) using Poisson regression.

**RESULTS:**

From the total of 9,412 individuals, 67.8% (95%CI 65.6–69.9) and 47.1% (95%CI 44.8–49.4) showed ≥ 2 and ≥ 3 diseases, respectively. In the adjusted analysis, women, older persons, and those who did not consume alcohol had increased multimorbidity. There were no associations with race, area of residence, geopolitical region, and coverage of the Family Health Strategy. The 10 pairs (frequencies observed between 11.6% and 23.2%) and the 10 triplets (frequencies observed between 4.9% and 9.5%) of the most frequent diseases mostly included back problems (15 times) and systemic arterial hypertension (11 times). All combinations were statistically higher than expected by chance.

**CONCLUSIONS:**

The occurrence of multimorbidity was high even among younger individuals (50 to 59 years). Approximately two in three (≥ 2 diseases) and one in two (≥ 3 diseases) individuals aged 50 years and over presented multimorbidity, which represents 26 and 18 million persons in Brazil, respectively. We observed high frequencies of combinations of morbidities.

## RESUMO

**OBJETIVO:**

Avaliar a ocorrência e os fatores associados à multimorbidade entre brasileiros com 50 anos ou mais de idade.

**MÉTODOS:**

Estudo transversal em uma coorte de base nacional da população brasileira não institucionalizada. Os dados foram coletados entre 2015 e 2016. A multimorbidade foi avaliada a partir de uma lista de 19 morbidades, sendo categorizada em ≥ 2 e ≥ 3 doenças. A análise incluiu cálculo de frequências e 10 pares e trios mais frequentes de combinações de doenças, além das análises bruta e ajustada dos fatores associados por meio de regressão de Poisson, incluindo variáveis demográficas, socioeconômicas, comportamentais e contextuais (zona de residência, região geopolítica e cobertura da Estratégia Saúde da Família).

**RESULTADOS:**

Do total de 9.412 indivíduos, 67,8% (IC95% 65,6–69,9) e 47,1% (IC95% 44,8–49,4) tinham ≥ 2 e ≥ 3 doenças, respectivamente. Na análise ajustada, mulheres, pessoas mais velhas e aqueles que não consumiam bebidas alcoólicas tiveram mais multimorbidade. Não foram observadas associações com cor da pele, zona de residência, região geopolítica e cobertura da Estratégia Saúde da Família. Os 10 pares (frequências observadas entre 11,6% e 23,2%) e os 10 trios (frequências observadas entre 4,9% e 9,5%) de doenças mais frequentes incluíram, em sua maioria, problema de coluna (15 vezes) e hipertensão arterial sistêmica (11 vezes). Todas as combinações apresentaram frequência estatisticamente maior do que seria esperado ao acaso.

**CONCLUSÕES:**

A ocorrência de multimorbidade foi elevada mesmo entre os indivíduos mais jovens (50 a 59 anos). Cerca de dois em cada três (≥ 2 doenças) e um em cada dois (≥ 3 doenças) indivíduos com 50 anos ou mais apresentaram multimorbidade, representando 26 e 18 milhões de pessoas no Brasil, respectivamente. Frequências elevadas de combinações de morbidades foram observadas.

## INTRODUCTION

Multimorbidity is the simultaneous occurrence of health problems in the same person, usually operationalized by the occurrence of ≥ 2 and ≥ 3 chronic diseases^1–3^. Recent studies have shown that multimorbidity is frequent in the world population and affects at least more than half of the older population^1^ ^,^ ^4^. It is an important public health problem because of its high frequency, especially because of its association with mortality, functional decline, and low quality of life, in addition to the difficulty of adequate management by health services^5–8^.

Studies in developed countries have shown a direct relation between socioeconomic indicators and prevalence of multimorbidity, which explains its relevance in the occurrence and maintenance of health inequities. A Scottish study has found a multimorbidity occurrence twice as high in the age group of 45–49 years for poorer individuals (26.8%) compared to the richest (13.4%). In addition, multimorbidity increased with age, and it reached 64.9% of the individuals aged between 65 and 84 years^4^.

Evidence of multimorbidity in low- and middle-income countries is still incipient in contrast to high-income countries^1^, despite the rapid increase in scientific research in recent years and the literature showing the higher occurrence of chronic diseases (in isolation) in developing countries^9^. In Brazil, despite some studies presenting results on the accumulation of diseases and their distribution by sociodemographic characteristics, only one study, carried out in a city in the South of Brazil, has evaluated the occurrence of multimorbidity specifically in the older population^10^. The findings showed a high occurrence of the problem in more than 60% of the population. It is more frequent among women, older persons, those with lower socioeconomic status, and those with less education, as well as among residents of areas covered by the Family Health Strategy (FHS).

Thus, a better understanding of the epidemiology of multimorbidity is necessary to subsidize policies within the Brazilian Unified Health System (SUS). This study aimed to estimate the prevalence and examine factors associated with multimorbidity among Brazilian individuals aged 50 years and over.

## METHODS

This is a cross-sectional study carried out with baseline data from the Brazilian Longitudinal Study of Aging (ELSI-Brazil), which was conducted with a national sample representative of the non-institutionalized population aged 50 years and over. The baseline study was conducted between 2015 and 2016. The sampling of the study was carried out by clusters combining the stratification of primary sampling units (municipalities), census tracts, and households. The municipalities were allocated in four strata according to population size. The method of Lavallée and Hidiroglou^11^ was used to decide the size and number of municipalities allocated in each stratum, which were: 1st) ≤ 26,700 inhabitants in 4,420 municipalities; 2nd) 26,701–135,000 inhabitants in 951 municipalities; 3rd) 135,001–750,000 inhabitants in 171 municipalities; 4th) > 750,000 inhabitants in 23 municipalities. For the first three strata, (municipalities up to 750,000 inhabitants), the sample was selected in three stages. In the first one, 18, 15, and 14 municipalities were selected for the first, second, and third strata, respectively. In the second stage, eight census tracts were selected in each municipality, and then the households were selected in each census tract in the third stage. In the fourth stratum, which included the largest municipalities, sample selection was carried out in two stages (1st – 176 census tracts were chosen; 2nd – households). Residents of the selected households aged 50 years and over were eligible for interviews. The final sample included 10,000 individuals aged 50 years and over (9,412 completed the interviews) living in 70 municipalities in different regions of Brazil. More details can be found on the research homepage^a^ and in a previous publication^12^.

The dependent variable was multimorbidity, measured by counting self-reported morbidities from a list of 19 diseases [(systemic arterial hypertension (SAH), back problems, high cholesterol, cataract, arthritis or rheumatism, depression, diabetes, osteoporosis, heart problem, glaucoma, emphysema, chronic bronchitis or chronic obstructive pulmonary disease (COPD), cerebrovascular accident, cancer, asthma, chronic renal failure, diabetic retinopathy, macular degeneration, Parkinson’s disease, and Alzheimer’s disease]. All of them were measured based on the interviewee’s report of the medical diagnosis of the disease: “Has any doctor told you that you have...?” Questions about eye problems (cataract, glaucoma, diabetic retinopathy, and macular degeneration) included the specialty of the ophthalmologist. More details on the measure can be obtained at the study website^a^. Multimorbidity was assessed according to two cutoff points: ≥ 2 and ≥ 3 morbidities^1^ ^,^ ^2^.

The independent variables included the following: sex (male, female); age in full years (50–59, 60–69, 70–79, 80 years and over); self-reported race (white, black, brown, yellow and indigenous, does not know or no answer); education level in years (zero, 1–4, 5–8, 9 years or more); index of household goods in quartiles constructed from the principal component analysis^13^ based on the number of household appliances, vehicles, and domestic workers at home; smoking (never smoked, current smoker – smokes daily or less than daily –, former smoker); consumption of alcoholic beverages (never, < once per month, ≥ once per month); area of residence (rural, urban); geopolitical region (North, Northeast, Southeast, South, Midwest); household covered by the Family Health Strategy (no, yes, does not know or no answer).

Data analysis was performed using the Stata program, SE 15.0. We carried out a descriptive analysis of the demographic, socioeconomic, behavioral, contextual, and morbidity variables. For each morbidity, we calculated the mean number of associated diseases. The general description and the description according to the independent variables of the occurrence of ≥ 2 and ≥ 3 morbidities included the calculation of percentage (%), 95% confidence interval (95%CI), and p value by the Pearson chi-square test. We calculated the occurrence of multimorbidity excluding SAH and high cholesterol from the list of morbidities. In addition, we performed crude and adjusted analyses using Poisson regression to evaluate the association between multimorbidity and all independent variables listed above. In these analyses, we obtained the prevalence ratios (PR), 95%CI, and p value (Wald test of heterogeneity). Associations that presented 95%CI without including nullity (PR = 1.00) and p value < 0.05 were considered statistically significant. For adjustment, all variables were considered at the same determination level, and we performed the selection by the backward elimination method. Thus, all variables were included in the model, excluding those with higher p value (Wald test of heterogeneity). Adjustment was performed until no variable with a p value > 0.20 remained in the model. The variables with p value > 0.20 are described in Table 4 with information on measures of effects, 95%CI, and p value of the model containing the variable, before it was excluded. To calculate the 10 pairs and 10 triplets of the most prevalent morbidities, we calculated the relation between the observed (O) and expected (E) values to measure the occurrence of combinations beyond that expected by chance^14^. The expected values were calculated by multiplying the individual prevalence of the diseases. The 95%CI of the O/E ratios were obtained through exact binomial probability. All analyses considered the sample design.

The ELSI-Brazil was approved by the Research Ethics Committee of the *Instituto René Rachou* of the Oswaldo Cruz Foundation (Protocol 886.754). All participants signed an informed consent form before the interviews. All regulatory and legal aspects were fulfilled.

## RESULTS

The final sample consisted of 9,412 individuals, and 54.0% were women and the mean age was 62.9 years. Approximately half of the sample was aged between 50 and 59 years and 7.1% was 80 years and over. More than 80% reported being white or brown. Approximately a quarter of the sample had nine or more years of schooling. Over a third (37.3%) consisted of former smokers. Most individuals (84.7%) lived in the urban area and the household was covered by the FHS (68.9%). Almost half (47.2%) lived in the Southeast region (Table 1). Individuals presented 2.66 morbidities on average. Arterial hypertension (52.2%), back problems (40.8%), and high cholesterol (30.5%) were the most frequent conditions, whereas macular degeneration (1.5%), Alzheimer’s disease (0.8), and Parkinson’s disease (0.7%) presented the lowest frequencies. The mean associated morbidities ranged from 3.5 for SAH to 5.9 for diabetic retinopathy (Table 2).

**Table 1:** Sample description according to independent variables. Brazilian Longitudinal Study of Aging (ELSI-Brazil), 2015–2016.

Variable	Category	n*	%
Sex	Female	5,314	54.0
Age (years)	50–59	3,980	47.6
	60–69	2,875	29.7
	70–79	1,782	15.6
	80 and over	775	7.1
Race	White	3,590	42.7
	Black	887	9.7
	Brown	4,283	44.7
	Yellow/Indigenous	310	2.9
	Does not know	342	3.4
Education level (years of study)	Zero	1,530	13.3
	1–4	3,638	38.2
	5–8	1,878	21.6
	9 or more	2,304	26.9
Index of goods (quartiles)	1st (lowest)	2,693	25.1
	2nd	2,421	24.9
	3rd	2,211	25.0
	4th (highest)	2,029	25.0
Smoking	Never smoked	4,259	45.6
	Current smoker	1,604	17.1
	Former smoker	3,546	37.3
Consumption of alcoholic beverages	Never	6,909	70.9
	< once a month	544	6.0
	≥ once a month	1,952	23.1
Area of residence	Urban	7,935	84.7
Geopolitical region	North	743	5.6
	Northeast	2,549	24.1
	Southeast	3,922	47.2
	South	1,278	16.6
	Midwest	920	6.6
Household covered by the FHS	No	2,358	25.8
	Yes	6,487	68.9
	Does not know/No answer	567	5.3

Total		9,412	100

FHS: Family Health Strategy

* Number of respondents without weighting.

**Table 2:** Description of morbidities and mean associated diseases. Brazilian Longitudinal Study of Aging (ELSI-Brazil), 2015–2016.

Morbidities	%	Mean associated morbidities
Systemic arterial hypertension	52.2	3.5
Back problems	40.8	3.9
High cholesterol	30.5	3.8
Cataract	24.9	4.0
Arthritis or rheumatism	21.0	4.5
Depression	18.6	4.5
Diabetes	15.8	4.2
Osteoporosis	15.8	4.8
Heart problem	11.7	4.8
Glaucoma	8.4	4.5
Emphysema, chronic bronchitis, or COPD	6.0	4.7
Cerebrovascular accident	5.3	4.6
Cancer	5.3	4.2
Asthma	4.9	4.8
Chronic kidney disease	4.5	5.1
Diabetic retinopathy	1.9	5.9
Macular Degeneration	1.5	5.3
Alzheimer’s disease	0.8	5.7
Parkinson’s disease	0.7	4.9

COPD: chronic obstructive pulmonary disease

We observed an increase in the number of morbidities according to age. While 17.7% of the individuals aged 50–59 years did not present any chronic morbidity, this value decreased to 2.7% among those aged 80 years and over. At the other extreme, 5.8% and 11.3% of the individuals aged 50–59 and 80 years and over, respectively, presented more than six morbidities (Figure).

**Figure:**
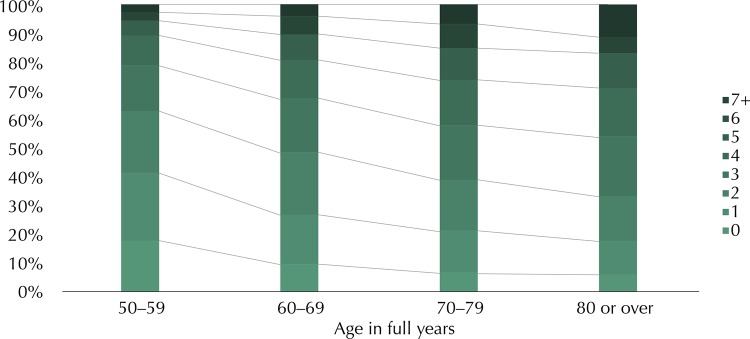
Occurrence of number of morbidities according to age group. Brazilian Longitudinal Study of Aging (ELSI-Brazil), 2015–2016.

The prevalence of multimorbidity was 67.8% (95%CI 65.6–69.9) for ≥ 2 and 47.1% (95%CI 44.8–49.4) for ≥ 3 diseases (both variables with a valid number of 8,848 individuals), and it was higher among women (20 percentage points higher for ≥ 3 morbidities), with increasing age, and among those who never consumed alcoholic beverages. No statistically significant differences were detected for the variables of race and FHS coverage (Table 3). When we excluded SAH and high cholesterol, the prevalences of ≥ 2 and ≥ 3 diseases were 49.0% (95%CI 46.7–51.2) and 29.3% (95%CI 27.4–31.2), respectively (data not presented in tables or figures).

**Table 3:** Prevalence of multimorbidity according to independent variables. Brazilian Longitudinal Study of Aging (ELSI-Brazil), 2015–2016.

Variable	Category	Multimorbidity			

		≥ 2		≥ 3	
	
		%	95%CI	%	95%CI
Sex			p < 0.001		p < 0.001
	Male	58.9	56.4–61.5	36.2	33.9–38.5
	Female	75.5	73.4–77.4	56.5	54.3–58.7
Age (years)			p < 0.001		p < 0.001
	50–59	58.8	56.1–61.5	37.1	34.5–39.7
	60–69	73.4	70.7–75.8	51.6	49.1–54.2
	70–79	79.0	75.9–81.7	61.1	57.3–64.7
	80 and over	82.4	78.5–85.7	66.7	62.1–71.0
Race			p = 0.112		p = 0.167
	White	68.5	65.7–71.2	48.4	45.7–51.1
	Black	71.4	65.8–76.4	50.1	44.1–56.1
	Brown	66.2	63.3–69.0	45.3	42.6–47.9
	Yellow/Indigenous	74.1	65.8–80.9	56.7	39.5–53.9
Education level (years of study)			p < 0.001		p < 0.001
	Zero	68.2	64.2–72.0	50.8	46.6–55.0
	1–4	71.9	69.2–74.4	50.4	47.5–53.3
	5–8	66.1	63.3–68.9	45.9	42.8–49.0
	9 or more	63.2	59.8–66.4	41.4	38.6–44.2
Index of goods (quartiles)			p = 0.010		p = 0.012
	1st (lowest)	64.0	61.0–67.0	44.6	41.1–48.1
	2nd	70.4	67.9–72.7	50.5	47.4–53.6
	3rd	69.1	65.3–72.6	48.2	45.2–51.3
	4th (highest)	67.7	64.4–70.9	45.0	41.5–48.5
Smoking			p < 0.001		p < 0.001
	Never smoked	68.4	66.0–70.7	48.2	45.6–50.8
	Current smoker	60.1	56.4–63.6	39.1	35.6–42.7
	Former smoker	70.6	67.9–73.1	49.4	46.7–52.0
Consumption of alcoholic beverages			p < 0.001		p < 0.001
	Never	71.3	69.1–73.4	51.4	49.0–53.8
	< once a month	63.5	57.9–68.8	43.1	38.1–48.3
	≥ once a month	58.5	55.6–61.4	35.3	32.5–38.3
Area of residence			p = 0.040		p = 0.083
	Rural	63.6	59.2–67.8	43.6	39.8–47.6
	Urban	68.8	66.1–70.9	47.7	45.1–50.3
Geopolitical region			p = 0.124		p = 0.130
	North	64.1	55.2–72.1	43.2	36.9–49.7
	Northeast	64.0	61.5–66.5	44.5	31.4–47.7
	Southeast	69.1	65.2–72.6	47.1	43.0–51.3
	South	70.7	65.7–75.2	51.7	48.0–55.3
	Midwest	69.0	63.2–74.2	48.3	42.7–54.0
Household covered by the FHS			p = 0.960		p = 0.639
	No	67.5	64.0–70.8	46.8	43.7–50.0
	Yes	67.9	65.2–70.5	47.0	44.2–49.7
	Does not know/No answer	68.0	62.0–73.4	50.1	44.6–55.5

Total		67.8	65.6–69.9	47.1	44.8–49.4

FHS: Family Health Strategy

In the crude analysis (for ≥ 2 or ≥ 3 morbidities), the variables of sex, age, race, education level, index of goods, smoking, and alcohol consumption were significantly associated with multimorbidity. After adjustment, women had 1.26 (95%CI 1.22–1.30) and 1.49 (95%CI 1.41–1.58) times more multimorbidity compared to men for the cutoff points of ≥ 2 and ≥ 3 chronic diseases, respectively. In relation to age, the occurrence of ≥ 3 diseases was 66% (95%CI 50–83) higher among individuals aged 80 years and over compared to those aged between 50 and 59 years. For ≥ 2 diseases, we observed a low magnitude association for education level; individuals with one to four years of schooling had 1.07 (95%CI 1.02–1.12) times more multimorbidity than those with no schooling. Respondents belonging to the lower strata of the index of goods presented less multimorbidity. Persons in the highest stratum of the index had 13% (for ≥ 2) and 12% (for ≥ 3) more multiple diseases than those in the lowest stratum. For education level and index of goods, we observed no dose-response relationship. Former smokers showed a higher occurrence of the outcome, in which it was at least 9% higher than those who had never smoked. Alcohol consumption equal to or greater than once a month was associated with a lower frequency of multimorbidity – 9% less for ≥ 2 and 17% less for ≥ 3 diseases compared to individuals who had never consumed these beverages (Table 4).

**Table 4:** Crude and adjusted analyses* between multimorbidity and independent variables. Brazilian Longitudinal Study of Aging (ELSI-Brazil), 2015–2016.

Variable	Multimorbidity							

	≥ 2				≥ 3			
	
	Crude analysis		Adjusted analysis		Crude analysis		Adjusted analysis	
			
	PR	95%CI	PR	95%CI	PR	95%CI	PR	95%CI
Sex (ref: male)								
Female	1.28	1.23–1.33	1.26	1.22–1.30	1.56	1.48–1.65	1.49	1.41–1.58
Age (ref: 50–59)								
60–69	1.25	1.19–1.31	1.22	1.17–1.28	1.39	1.29–1.50	1.36	1.26–1.46
70–79	1.34	1.27–1.41	1.30	1.23–1.37	1.65	1.51–1.80	1.56	1.43–1.69
80 and over	1.40	1.31–1.50	1.34	1.26–1.43	1.80	1.64–1.97	1.66	1.50–1.83
Race (ref: white)								
Black	1.04	0.96–1.14	1.08	0.99–1.17	1.03	0.92–1.16	1.07	0.98–1.18
Brown	0.97	0.93–1.01	1.01	0.97–1.05	0.93	0.88–0.99	0.99	0.93–1.04
Yellow/Indigenous	1.08	0.97–1.21	1.10	0.99–1.21	0.96	0.83–1.12	0.96	0.83–1.12
Education level (ref: zero)								
1–4	1.05	1.00–1.11	1.07	1.02–1.12	0.99	0.92–1.07	1.04	0.97–1.12
5–8	0.97	0.91–1.03	1.04	0.98–1.10	0.90	0.82–0.99	1.04	0.95–1.14
9 or more	0.93	0.86–1.00	0.97	0.91–1.05	0.81	0.74–0.90	0.93	0.85–1.03
Index of goods (ref: 1st)								
2nd	1.10	1.04–1.16	1.10	1.05–1.15	1.13	1.03–1.24	1.13	1.04–1.24
3rd	1.08	1.01–1.15	1.10	1.04–1.16	1.08	0.99–1.18	1.11	1.02–1.23
4th (highest)	1.06	0.99–1.13	1.13	1.07–1.19	1.01	0.91–1.12	1.12	1.01–1.24
Smoking (ref: never smoked)								
Current smoker	0.88	0.83–0.93	0.97	0.92–1.02	0.81	0.75–0.87	0.97	0.90–1.04
Former smoker	1.03	1.00–1.07	1.09	1.06–1.13	1.02	0.98–1.07	1.13	1.08–1.18
Consumption of alcoholic beverages (ref: never)								
< once a month	0.89	0.82–0.97	0.93	0.86–1.02	0.84	0.74–0.94	0.92	0.82–1.02
≥ once a month	0.82	0.79–0.86	0.91	0.87–0.95	0.69	0.64–0.74	0.83	0.77–0.89
Area of residence (ref: rural)								
Urban	1.08	1.00–1.16	1.05	0.99–1.12	1.09	0.99–1.21	1.09	1.00–1.18
Geopolitical region (ref: North)								
Northeast	1.00	0.87–1.15	0.98	0.86–1.11	1.03	0.87–1.22	0.99	0.86–1.14
Southeast	1.08	0.93–1.25	1.04	0.91–1.18	1.09	0.92–1.30	1.04	0.90–1.20
South	1.10	0.95–1.28	1.06	0.92–1.22	1.19	1.01–1.41	1.13	0.97–1.31
Midwest	1.08	0.93–1.25	1.05	0.93–1.19	1.12	0.93–1.34	1.08	0.94–1.24
Household covered by the FHS (ref: no)								
Yes	1.01	0.95–1.07	1.01	0.95–1.07	1.00	0.93–1.08	0.98	0.92–1.06
Does not know/No answer	1.01	0.91–1.11	0.98	0.88–1.08	1.07	0.93–1.22	1.01	0.88–1.17

FHS: Family Health Strategy

* Adjustment performed for all independent variables in the table.

The 10 pairs and triplets of the most frequent diseases mostly included back problems (15 times) and SAH (11 times). For the pairs, we could observe that one in four presented SAH and back problems at the same time and one in five had SAH and high cholesterol together. For the triplets, practically one in 10 presented SAH, back problems, and high cholesterol and SAH, back problems, and associated arthritis or rheumatism. All 10 most prevalent pairs and triplets had a higher than expected occurrence by chance (Table 5).

**Table 5:** Occurrence of the 10 pairs and triplets of the most frequent morbidities. Brazilian Longitudinal Study of Aging (ELSI-Brazil), 2015–2016.

Pairs and triplets of morbidities	Observed (O)	Expected (E)	O/E	95%CIz
	
	%	%		
Pairs				
SAH/Back problems	23.2	21.3	1.09	1.06–1.12
SAH/High cholesterol	18.9	15.9	1.19	1.15–1.23
SAH/Cataract	14.8	13.0	1.14	1.09–1.18
Back problems/High cholesterol	14.8	12.4	1.19	1.14–1.24
Back problems/Arthritis or rheumatism	14.5	8.6	1.69	1.62–1.77
Back problems/Cataract	12.0	10.2	1.18	1.13–1.23
SAH/Arthritis or rheumatism	12.3	11.0	1.12	1.07–1.17
Back problems/Osteoporosis	11.2	6.4	1.74	1.65–1.83
SAH/Depression	11.4	9.7	1.17	1.12–1.23
Back problems/Depression	11.6	7.6	1.53	1.46–1.60
Triplets				
SAH/Back problems/High cholesterol	9.5	6.5	1.46	1.39–1.54
SAH/Back problems/Arthritis or rheumatism	8.6	4.5	1.93	1.81–2.05
SAH/Back problems/Cataract	7.5	5.3	1.41	1.33–1.49
SAH/Back problems/Depression	7.2	3.4	1.81	1.70–1.93
SAH/Back problems/Osteoporosis	6.7	4.0	1.99	1.85–2.13
Back problems/Arthritis or rheumatism/Osteoporosis	6.5	1.4	4.76	4.32–5.26
Back problems/High cholesterol/Arthritis or rheumatism	5.8	4.0	2.21	2.05–2.39
Back problems/Arthritis or rheumatism/Depression	5.8	2.6	3.63	3.30–3.98
SAH/High cholesterol/Cataract	5.4	1.6	1.36	1.27–1.46
Back problems/Cataract/Arthritis or rheumatism	4.9	2.1	2.28	2.10–2.49

SAH: systemic arterial hypertension

## DISCUSSION

The results of the analysis show that the occurrence of multimorbidity was high even in the younger age groups. Of the total, two out of three and one in two individuals presented ≥ 2 and ≥ 3 diseases, respectively, confirming the relevance and extent of multimorbidity in the Brazilian older population. Women, older individuals, and those who never consumed alcoholic beverages showed the highest prevalence of multimorbidity. In addition, the 10 pairs and triplets of the most frequent diseases showed the relevance of combinations of morbidities of different systems in the organism.

According to data from the last population census, Brazil had approximately 39 million individuals aged 50 years and over. It is estimated that approximately 26 and 18 million persons have ≥ 2 and ≥ 3 morbidities, respectively. The adequate management of these individuals is a complex challenge and it will require the planning and articulation of actions from the SUS to meet this demand.

The prevalence found for multimorbidity is similar to that observed in international studies. The prevalence of ≥ 2 morbidities in our study was approximately 70%, which is similar to the population-based studies included in a systematic review^1^. A recent article included the occurrence of multimorbidity in nine countries, and it covered studies with data collection between 2007 and 2012 using the 12 most prevalent chronic conditions. The highest frequency of ≥ 2 diseases was observed in Russia (71.9%) and the lowest were in China (45.1%) and Ghana (48.3%). When considering the confidence interval of our study, the Brazilian findings are the same as those found in Spain, Finland, Poland, and Russia^15^. Thus, an occurrence of multimorbidity in Brazil similar to high-income countries can be observed, despite important socioeconomic differences. The demographic and epidemiological transition in the country has occurred faster than that observed in other countries, especially in developed countries. Thus, with the aging of the population, chronic non-communicable diseases become highly frequent problems with different negative impacts on the health of individuals. This situation challenges Brazilian health managers, policy makers, and scientists to improve the living conditions and the health system for the care of older adults with multiple needs, going beyond the existing actions to address these morbidities^16^. The comparison of evidence of ≥ 3 diseases with other studies presented a pattern similar to that observed for ≥ 2 morbidities.

The methodological differences between multimorbidity studies hinder a comparison with the findings. Notwithstanding, the recent efforts to standardize the concept^17^ and the operationalization of the construct^1^, there is still no universal definition for the evaluation of the subject. The difficulty of standardization may be explained given the different existing morbidities, the heterogeneous patterns of occurrence of diseases among countries, and the variable relevance of acute and chronic conditions in the different contexts. Nonetheless, a consensus is needed to improve the understanding of multimorbidity, or at least a standardized list of morbidities for each country.

The inclusion of conditions which are not diseases themselves is an important issue to be included in this debate. For example, SAH and high cholesterol are commonly used conditions to measure multimorbidity^1^. Nevertheless, both are considered risk factors for chronic non-communicable diseases^18^ ^,^ ^19^ and tend to inflate the occurrence of multimorbidity because of their high frequencies. In our sample, when we excluded SAH and cholesterol from the multimorbidity construct, the prevalence of the outcome decreased by approximately 18 percentage points for both ≥ 2 and ≥ 3 morbidities. Evaluations should be carried out regarding the importance and utility of including these conditions in multimorbity analysis.

Among the factors associated with the outcome, the female sex is usually associated with the greater occurrence of multiple problems in older adults^20^. This result is explained by the survival bias, since men have a shorter life expectancy, and survivors tend to have fewer health problems^21^. In addition, women are more likely to use health services^22^, and they are thus more likely to be diagnosed with diseases. The association with age may be explained by the greater exposure to stressful events throughout life, which compromise the physiological balance and facilitate the onset of chronic diseases^20^.

The greater occurrence of multimorbidity in the strata of higher index of goods diverges, to a certain extent, from other Brazilian studies. A national study carried out with adults^23^ and local studies with adults and older adults^10^ ^,^ ^24^ have observed similarities in the occurrence of the problem according to an inverse pattern or social stratum – economically unfavorable individuals presented more multimorbidity. Despite the differences in the used indicators of socioeconomic level, this result deserves attention and a more detailed analysis for a better understanding of the association. International evidence tends to show a higher incidence of diseases in poorer individuals^4^ ^,^ ^20^, which confirms the social determination in the health-disease process. An explanation for the result of our study is related to the fact that individuals from more favorable economic strata have greater access to health services and, consequently, are more frequently diagnosed with morbidities. The information on the evaluated morbidities was obtained by the interviewee’s report on the medical diagnosis of the disease, which is strongly associated with access to health services. In addition, this indicator presents an important relation with education level (usually associated with multimorbidity, despite the lack of association in the adjusted analysis of our study). We observed that individuals from the lowest stratum of the index of goods showed a lower occurrence of multiple problems, especially in the adjusted analysis. When we verified the variable that most explains this increase in the effect of the index of goods after adjustment (data not shown), it is evident that the inclusion of education level in the model increases the strength of the association. Education level is an important factor in the understanding of older adults about their existing health condition and diseases^25^. Finally, the result can be affected by the lack of fit to the variables of use of services or residual confusion. The increased understanding of these relations, including issues related to the use of health services and coverage of care models, may elucidate the observed results, mainly considering the important existing regional differences in Brazil.

Former smokers tend to present early health problems^26^ and, consequently, greater occurrence of multimorbidity. In addition, these individuals have a higher risk of death, which may explain the association of low magnitude (between 9% to 13% higher in relation to those who had never smoked). This result can be explained by the survival bias; survivors are healthier, which attenuates the association found.

The lower frequency of multiple problems among individuals who consumed alcoholic beverages (one or more times per month) can be explained by the reverse causality bias given the cross-sectional evaluation performed in this article. Individuals who reported some alcohol consumption may have fewer morbidities or the fact that they have fewer diseases could lead to higher alcohol consumption . Thus, it is not yet possible to define temporality in the association. Longitudinal assessments of the study, which include incident cases, may contribute to assess possible causal relations between these variables.

The pairs and triplets of diseases showed high frequencies of co-occurrence of morbidities with emphasis on the presence of SAH and back problems in most combinations. These analyses contribute to the creation of protocols and clinical guidelines for the health care of older adults. Almost a quarter of the evaluated individuals presented SAH and back problems, thus showing that the management of these individuals would need an integral analysis to treat the conditions. If physical activity is recommended for blood pressure control, back problems must be also considered, so that the management of one condition does not affect other morbidity^27^. In addition, as we evaluated frequent conditions, these morbidities may occur concomitantly because of chance. Thus, we calculated the relation between the occurrence observed and the one expected by chance in the pairs and triplets. All patients had a ratio greater than one, and this suggests that morbidities have some type of relation, whether causal, if one disease or its treatment increases the risk of another, or non-causal, if the morbidities have common risk factors^14^.

The study presents limitations which should be considered. First, multimorbidity was evaluated from a list of diseases and the interviewee’s report of medical diagnosis, which has a strong relation with access to health services and may reduce the occurrence of multimorbidity in less favored socioeconomic groups. Second, the measurement of some diseases by medical diagnosis has limitations related to the low sensitivity and specificity of this type of data collection^25^, especially for mental health problems^28^. New analyses on multimorbidity should include measures of morbidity with greater diagnostic ability. In addition, validation studies on the measurement of self-reported medical diagnosis should be performed for the clear understanding of the diseases which may be included in the multimorbidity construct. Third, the cross-sectional analysis hinders the evaluation of some associations, such as between smoking and alcohol consumption and multimorbidity, as there may be reverse causality.

The findings on multimorbidity are an important baseline for future longitudinal analyses which evaluate the impact of multiple diseases on health outcomes and quality of care^29^. To ensure a better quality of life for Brazilian older adults we need to act under a comprehensive health assessment, which, in turn, demands an understanding of the occurrence and impact of multimorbidity. The results show high occurrence of multimorbidity and the factors associated with the problem. The pairs and triplets identified show the main combinations of diseases found in the population aged 50 and over, which can be included in clinical guidelines and evaluated in the care provided in the health services.

## Footnotes

a Fundação Oswaldo Cruz. Brazilian Longitudinal Study of Aging. Rio de Janeiro; c2015 [cited 2017 Nov 28]. Available from: http://elsi.cpqrr.fiocruz.br
